# A Treatise on Food and Diet, with Observations on the Dietetical Regimen Suited for the Disordered State of the Digestive Organs, &c.

**Published:** 1844-05

**Authors:** 


ART. VIII. *A Treatise on Food and Diet, with Observations on the Dietetical Regimen suited for the Disordered States of the Digestive Organs; with an account of the Dietaries of some of the principal Metropolitan and other establishments for Paupers, Lunatics, Criminals, Children, the Sick, &c.* By JONATHAN PERIERA, M. D. F. R. S., and L. S. &c. &c. Edited by CHARLES A. LEE, M. D. New York: J. and H. Langley. 1843.

In a former article we gave some account of the first two chapters of this work, which treat of *the elements of food*, and *alimentary principles.* We propose now completing our notice of it, by a brief survey of the remaining topics.

All the articles which nourish man are derived from the organic kingdom. He requires a mixed diet of animal and vegetable food. This is proved by the structure of his teeth and of the entire human digestive apparatus, as well as by the fact that the most perfect development and vigor of mind and body are displayed by the nations in which such a diet prevails. The long catalogue of these articles is given by Pereira in his third chapter, on *compound aliments*, which embraces, necessarily, a multitude of details, only a few of which we shall be able to copy; and in doing this we shall endeavor to select such as are most recommended by novelty, or their practical bearing. In connexion with the nutritive qualities of bones, he relates the following incidents, which are instructive as well as amusing:

"At the commencement of the French revolution, the attention of every one in France was directed to the improvement of the food for the poor and for the army. All were agreed in employing for this purpose bones. The government, led away by the enthusiastic reports of scientific men, (Proust, d'Arcet, Pelletier, Cadet de Vaux, &c.,) issued a public instruction, declaring that "a bone is a tablet of soup formed by nature: a pound of bone gives as much soup as six pounds of meat: bone soup, in a dietetical point of view, is preferable to meat soup." It need scarcely be stated that these inflated expressions were gross exaggerations. It is obvious, as Magendie has justly observed, that in this hyperbolic language the terms *jelly* (gelatine) and *nutritive matter* were considered synonymous. The favorable report made by the Faculty of Medicine, on the nutritive and easily digestible properties of gelatine, induced the French *administration des hospices*, in 1824, to introduce its employment into the public hospitals of Paris; and for this purpose, in many of these establishments d'Arcet's apparatus for obtaining a solution of gelatine from bones by the aid of steam, was fitted up. In most, if not in all, its employment was, however, soon abandoned."

His remarks on milk, as an article of diet, will be read with interest. It may not be known to every one that tubercular phthisis frequently affects cows, and that the milk of such animals is degenerated from its healthy state. We have witnessed this disease in a number of cows which were imported from Ireland, and in some assured ourselves of its character by *post mortem* examinations. These revealed the most extensive deposits of tuberculous matter and general disorganization of the lungs:

"The quality of the milk is also affected by the state of health of the female supplying it. I have already mentioned the effect of tubercular disease of the lungs in increasing the quantity of phosphate of lime in the milk. This subject is one of the greatest moment, not only in reference to the frequency of disease in cows, and, therefore, to the possible morbific character of their milk, but also in reference to the milk of the human subject. I think, with these facts before us, it would be highly improper to allow a female, with any trace or suspicion of tuberculous disease, to suckle. Not that a few grains, more or less, of phosphate of lime in the milk, can probably do any injury to the child; but the fact once established, that the milk may be thus altered by disease, leads to the suspicion that some other substances, not yet recognized by their physical or chemical characters, may be in the milk of diseased nurses, and which may have an injurious influence on the child; and the suspicion does not confine itself to those affected with tuberculous diseases: other hereditary or constitutional affections may also be attended with altered conditions of the milk. This suspicion is strengthened by the common observation that the milk of different nurses does not equally suit the same child; nor that of the same nurse, different children."

The following statistics of the editor, Dr. Lee, belong rather to political economy than to medicine, but we are sure our readers will be obliged to us for the quotation of them:

"Indian corn and potatoes, indigenous to our country, have contributed much to promote the health and longevity of mankind in both hemispheres. As they are among the cheapest, so also are they among the most wholesome of all articles of food employed by man. Good corn weighs about 60 lbs. to the bushel, and costs at present 56 cents per bushel, or nearly one cent per pound. Now a pound of corn, when cooked, makes from two and a half to three and a half pounds of food, and this will suffice for the daily support of a laboring man. If an individual could be supported on this alone, his annual expense for food would be but $3 65, or say $15 to a family of five. The average cost of potatoes may be put at about half a cent a pound, and allowing five pounds per day to an adult individual, the expense will be about $9 a year; or for a family of five, (reckoning them at three and a half adults,) about $30. When we consider that it is not unusual for land to yield 100 bushels of corn to the acre, and 30 tons, or 67,200 pounds of potatoes to the acre, we may form some estimate of the population which this country is capable of supporting from the produce of the soil."

Have any of our readers lived to this time without being acquainted with the surpassing excellences of the *corn pudding?* We have supposed such a thing possible, and here pause in our grave task of reviewing this instructive book, to exhort such as are yet strangers to the merits of this most delicious of puddings, to procure the recipe for making it before the next "roasting ear" season. The corn being well grated, is mixed with butter, eggs and milk, seasoned with salt and pepper, and thoroughly baked. Thus prepared, no dyspeptic need fear it, and no man who is not indifferent to the good things of this world will sit down to dinner without one of these puddings on his table, during the season of young corn.

Our author esteems potatoes highly for their antiscorbutic properties. In prisons, where men are confined to few articles of food, scurvy frequently breaks out. It has been found that the disease is prevented by adding a few pounds of these tubers to the weekly rations of the convicts. He makes the following statement in regard to the effect of freezing upon the potato, which, we dare say, will be new to most of our readers:

"The influence of a freezing temperature on the potato is remarkable. The effect is mechanical; the watery juice contained in the cells and intercellular spaces, expands in the act of freezing, and by this means ruptures and isolates the cells, and destroys the organization of the tuber. It does not appear, however, that any chemical change is produced in the first instance either on the starch or the other constituents, for Girardin obtained the same proportions of water, fecula, woody fibre, albumen, sugar, and saline matters, from frosted, as from unfrosted potatoes. But it is obvious that when the organization and life of the potato is destroyed, decomposition must soon succeed; though even then the fecula or starch seems but little altered."

The reader will find every thing in this part of Dr. Pereira's work that he can wish to know, concerning "solid foods, drinks, and condiments." We only select a remark as we pass along, which may serve as a specimen. Of bread he says:

"Notwithstanding that bread is denominated the *staff of life*, alone it does not appear to be capable of supporting prolonged human existence. Boussingault came to this conclusion from observing the small quantity of nitrogen which it contains; and the Reports of the Inspectors of Prisons, on the effects of a diet of bread and water, favor this notion."

Meat alone, on the contrary, does appear to be sufficient. Dr. Lee, in his appendix to this work, cites some facts going to establish it. Thus:

"Sir Francis Head relates some interesting particulars respecting the Gauchos, inhabitants of the Pampas, in South America, which have an important bearing on this question. After stating that they often continue on horseback day after day, galloping over their boundless plains, under a burning sun, and performing labors almost of an incredible description, he remarks: 'As the constant food of the Gauchos is *beef and*

*water*, his constitution is so strong, that he is able to endure great fatigue, and the distances he will ride, and the number of hours he will remain on horseback, would hardly be credited.' Sir Francis Head also brings his own personal experience in proof of the correctness of the above statement.

"'When I first crossed the Pampas,' he remarks, 'I went with a carriage, and although I had been accustomed to riding all my life, I could not at all ride with the Peons, (drivers of the carriage,) and after galloping five or six hours, was obliged to get into the carriage; but after I had been riding for three or four months, and had lived upon beef and water, I found myself in a certain condition, which I can only describe by saying that I felt no exertion could kill me. For a week I could daily be upon my horse before sunrise, could ride till two or three hours after sunset, and have really tired out ten or twelve horses. This will explain the immense distances which people in South America are said to ride, which I am confident could only be done on beef and water.' "—*Rough Notes, &c., by Sir Francis Head*, p. 29.)

This does not harmonize exactly with the assertion we made at the beginning of this article, on the authority of physiologists, that "the most perfect physical development, and the greatest intellectual vigor are to be found among those races in which a mixed diet is the prevalent habit." Still there can be no question of the truth of this assertion. The peculiar habits of these Tartars of South America render the exclusive diet of animal food congenial to their systems, but if adopted generally by men, it would prove ruinous to health and strength of constitution. It is only persons of the most laborious habits who can with impunity partake largely in hot weather of animal food.

In the second part of his work, Dr. Pereira treats of *Diet*—the digestibility and nutritive qualities of foods, times of eating, dietaries, and dietetical regimen for the sick.

On the question of repose, or activity after eating, he expresses the following opinion:

"These and many other analogous facts are satisfactory to my mind that repose is natural to animals after a hearty meal; and that the practice of taking the *siesta*, or after dinner sleep, is not injurious, if moderately indulged in. It should, however, be followed by moderate exercise. But there are exceptions to these statements, and I have met with some few persons who have asserted that they find advantage in using exercise immediately after dinner; but these form exceptions to the general rule. After the earlier and lighter meals of the day, breakfast or luncheon, quietude or repose is neither desired nor required."

Of the times of eating, he says:

"These facts show the importance of breakfasting soon after rising and dressing; at least in many cases. I am fully aware that there are numerous exceptions to this. Some persons not only suffer no injury from, but actually appear to be benefited by, active exercise taken before breakfast; its effect being with them to create or augment the appetite. But in others the effects are those which I have already stated. I am satisfied, from repeated observation, that in children disposed to spasmodic and other brain diseases, the practice of making them attend school for two hours before breakfast is injurious; and I fully agree, therefore, with Dr. Combe, that in 'boarding-schools for the young and growing, who require plenty of sustenance, and are often obliged to rise early, an early breakfast is almost an indispensible condition of health.' Epileptics, especially those disposed to morning attacks, should invariably breakfast soon after rising. I think I have seen the fits brought on by neglecting this precaution. For travellers a light breakfast before starting is a great protection 'against colds and subsequent fatigue or exhaustion.' Medical men and others should not, if possible, expose themselves to the influence of infectious or contagious disorders, as fevers, &c., before breakfast, as the danger of infection then is greatly enhanced. For the same reason the practice of making post-mortem examinations and dissections before breakfast is to be condemned."

Concerning *dietaries for children* he has the following sensible and pleasant remarks:

"In children the function of nutrition is more active than in adults. They have not merely to repair the daily waste, that is, to renovate their tissues, but to grow. Their functions of circulation and respiration are, therefore, more active than in after life; and they require food; that is, substances to support the process of respiration, to be administered at shorter intervals."

"Children, for the most part, evince an almost instinctive fondness for sugar, which is supplied to them in their mother's milk. This perhaps is to be explained by the fact that it is an element of respiration, and, therefore, is more necessary for them than adults, on account of the greater activity of their function of respiration. But this fondness for sugar is by no means universal among children. In very cold countries, substances richer in carbon and hydrogen, and, therefore, yielding more heat by combustion, are preferred. 'In one of those late extravagant voyages to discover a northwest passage,' says Sir Anthony Carlisle, 'the most northern race of mankind were found to be unacquainted with the taste of sweets, and their infants made very wry faces, and sputtered out sugar with disgust; but the little urchins grinned with ecstacy at the sight of a bit of whale's blubber.'

The natural appetite I believe to be an index of the wants of the system; and ought, therefore, to be consulted, to a certain extent, in the dieting of children; and I believe that parents commit a gross error who totally disregard it. I have seen children refused vegetable food, though they ardently desired it, because they would not eat what their nurses supposed to be the proper proportion of animal food; and, on the other hand, I have known children denied animal food, on the mistaken notion that it would be injurious to them, though the digestive functions were active, and the appetite for meat most keen."

"Children may be over fed or under fed. Instances of the former, however, are comparatively rare. Of the ill consequences of defective nutriment we have, unfortunately, too many instances continually presented to our notice. Irritable bowels or diarrhœa, tumid abdomen, mesenteric disease, wasting, and fever, are the ordinary and obvious effects. They frequently follow the continued use of pea-soup and potato-stews—dishes which are in common use at poor-houses and other establishments for pauper children. Scrofulous and strumous diseases, marasmus, rickets, distortions, and pot bellies, so commonly met with among children of the poor, are referrible, in part, at least, to food, defective either in quantity or quality, or perhaps in both. I think it will be found that more than two-thirds of pauper children are strumous. They derive this condition in part, perhaps, from hereditary tendency; but partly also, as I believe, from defective nutriment,. To the same cause, also, is ascribable their inferior developement. If the children in poor-houses be examined, they will be found, for the most part, smaller and shorter for their age, more frequently distorted, and more readily fatigued, than the children of the middling and higher classes."

The section on *dietaries for the sick* abounds, as does indeed every portion of the work, in details of uncommon interest for the practitioner of medicine. The fever diet, the low diet, the animal diet, the vegetable diet, and all the other modifications demanded in man's aliment by his ever-varying disorders, are here given with all desirable minuteness. On this subject we will give but a single passage, which our author quotes with approbation from Dr. Billing:

"Natural instincts," justly observes Dr. Billing, "are too often thwarted: it is much too common to put patients empirically on low diet; and patients of the higher classes—the better educated—very often put themselves on low diet unnecessarily. So far as we may take natural instinct for a guide," he further observes, "we may assert, that when a patient *can* eat, he may be allowed to do so; for if he has even a slight degree of fever, he *cannot* eat."

The Appendix, by Dr. Lee, supplies the omissions of the author, and adds to the value of the treatise. We take leave of it by again recommending it to those who wish an entertaining volume on a subject which comes home, if not to "the business and bosoms," at least to the "stomachs and interests" of all classes of men. Y.

